# MendelianRandomization v0.9.0: updates to an R package for performing Mendelian randomization analyses using summarized data

**DOI:** 10.12688/wellcomeopenres.19995.2

**Published:** 2023-11-21

**Authors:** Ashish Patel, Ting Ye, Haoran Xue, Zhaotong Lin, Siqi Xu, Benjamin Woolf, Amy M. Mason, Stephen Burgess

**Affiliations:** 1 MRC Biostatistics Unit, University of Cambridge, Cambridge, England, CB2 0SR, UK; 2 Department of Biostatistics, University of Washington, Seattle, Washington, USA; 3 Department of Biostatistics, City University of Hong Kong, Hong Kong, Hong Kong; 4 Division of Biostatistics, School of Public Health, University of Minnesota Duluth, Duluth, Minnesota, USA; 5 Department of Statistics, Florida State University, Tallahassee, Florida, USA; 6 Department of Statistics and Actuarial Science, The University of Hong Kong, Hong Kong, Hong Kong; 7 Medical Research Council Integrative Epidemiology Unit, University of Bristol, Bristol, England, UK; 8 School of Psychological Science, University of Bristol, Bristol, England, UK; 9 British Heart Foundation Cardiovascular Epidemiology Unit, Department of Public Health and Primary Care, University of Cambridge, Cambridge, England, UK; 10 Victor Phillip Dahdaleh Heart and Lung Research Institute, University of Cambridge, Cambridge, England, UK

**Keywords:** Mendelian randomization, instrumental variable, summarized data, genetic epidemiology, post-GWAS analysis, causal inference, genetic associations

## Abstract

The MendelianRandomization package is a software package written for the R software environment that implements methods for Mendelian randomization based on summarized data. In this manuscript, we describe functions that have been added or edited in the package since version 0.5.0, when we last described the package and its contents. The main additions to the package since that time are: 1) new robust methods for performing Mendelian randomization, particularly in the cases of bias from weak instruments and/or winner’s curse, and pleiotropic variants, 2) methods for performing Mendelian randomization with correlated variants using dimension reduction to summarize large numbers of highly correlated variants into a limited set of principal components, 3) functions for calculating first-stage F statistics, representing instrument strength, in both univariable and multivariable contexts, and with uncorrelated and correlated genetic variants. We also discuss some pragmatic issues relating to the use of correlated variants in Mendelian randomization.

## Introduction

Assessing causality in relationships between risk factors and outcomes is tricky and subject to many potential pitfalls. Mendelian randomization is an epidemiological technique that assesses such questions using genetic variants ^1, 2^. Mendelian randomization uses genetic variants as instrumental variables to assess evidence for the causal effect of an exposure on an outcome.

An instrumental variable is a variable that behaves analogously to randomization in a randomized trial. It divides the population into groups with different distributions of the exposure, but the groups are otherwise indistinguishable (aside from any differences occurring due to downstream causal effects of the exposure) ^3^. Formally, an instrumental variable should be associated with the exposure (relevance), not associated with the outcome via any confounding pathway (exchangeability), and does not affect the outcome directly, only potentially indirectly via an effect of the exposure (exclusion restriction) ^4^. Generally speaking, genetic variants are plausible candidate instrumental variables, as they tend to have specific effects on particular biological mechanisms, they are uncorrelated with genetic variants that influence other biological mechanisms (due to Mendel’s laws of inheritance), and they are fixed at conception (hence not subject to reverse causation or influence by environmental factors) ^5^.

MendelianRandomization is a software package written for the R software environment ^6^ that implements methods for Mendelian randomization based on summarized data ^7^. By summarized data, we mean genetic associations with traits (beta-coefficients and standard errors) taken from regression analyses of a trait on a genetic variant ^8^. Such associations are estimated in genome-wide association studies. They have been publicly reported for millions of variants by many large studies and consortia, and can be accessed through several different public portals ^9, 10^.

This package has previously been introduced ^11^, and updates up to version 0.5.0 have been discussed ^12^. In this paper, we present updates to the package since version 0.5.0 up to the current version 0.9.0. A complete list of functions in the package is given in Table 1. The properties of the various methods are not discussed here in detail; we encourage users to read the relevant references for the specific methods or the guidelines paper for general advice on performing Mendelian randomization investigations ^13^. We also encourage users to consult the documentation provided with the package, which describes all the options available for each method in greater detail. In this paper, we aim to provide an overview of recent updates to the package.

**Table 1.** Functions available in the MendelianRandomization package. Functions are divided into five categories: data entry functions, univariable estimation methods, multivariable estimation methods, data visualization functions, and functions that load data from PhenoScanner.

Function	Description	Status	Can include correlated variants?
Data entry functions			
mr_input	Data entry for univariable analysis		
mr_mvinput	Data entry for multivariable analysis		
Univariable estimation methods			
mr_ivw	Inverse-variance weighted (IVW) method	†	✓
mr_median	Median method		
mr_egger	MR-Egger method		✓
mr_maxlik	Maximum likelihood method		✓
mr_mbe	Mode-based estimation method		
mr_hetpen	Heterogeneity penalized method		
mr_conmix	Contamination mixture method		
mr_lasso	Lasso method		
mr_cML	Constrained maximum likelihood method	*	
mr_divw	Debiased inverse-variance weighted method	*	
mr_pivw	Penalized inverse-variance weighted method	*	
mr_pcgmm	Principal component generalized method of moments method	*	✓
mr_allmethods	Runs several methods		
Multivariable estimation methods			
mr_mvivw	Multivariable IVW method	†	✓
mr_mvmedian	Multivariable median-based method		
mr_mvegger	Multivariable MR-Egger method		✓
mr_mvlasso	Multivariable lasso method		
mr_mvcML	Multivariable constrained maximum likelihood method	*	
mr_mvgmm	Multivariable generalized method of moments method	*	✓
mr_mvpcgmm	Multivariable principal component generalized method of moments method	*	✓
Data visualization functions			
mr_plot	Scatter plot		
mr_forest	Forest plot		
mr_funnel	Funnel plot		
mr_loo	Leave-one-out plot		
Loading data from PhenoScanner			
extract.pheno.csv	Data entry from PhenoScanner .csv file (legacy)		
pheno_input	Data entry from web-based PhenoScanner		

* = new since version 0.5.0, † = updated since version 0.5.0, ✓ = estimation method allows variants to be correlated (if not, then the method assumes variants are uncorrelated).

## Methods

### Implementation

***Constrained maximum likelihood methods.*** The mr_cML and mr_mvcML functions perform the constrained maximum likelihood method, for the univariable Mendelian randomization ^14^ ( mr_cML) and multivariable Mendelian randomization ^15^ ( mr_mvcML) settings. These methods are robust to the violation of any of the three instrumental variable assumptions under mild assumptions. In a maximum likelihood framework, these methods constrain the number of invalid instruments with horizontal pleiotropy. The number of invalid instruments is asymptotically consistently selected by the Bayesian information criterion. To further account for the selection uncertainty with a finite sample, a data perturbation approach is employed.

The mr_cML function takes an *MRInput* object as input, created using the mr_input command. The syntax is:


mr_cML(mr_input(ldlc, ldlcse, chdlodds, chdloddsse),n=17723)


where ldlc and ldlcse are genetic associations with low-density lipoprotein (LDL) cholesterol and their standard errors for 28 uncorrelated genetic variants previously reported as associated with at least one of LDL-cholesterol, high-density lipoprotein (HDL) cholesterol, or triglycerides by Waterworth *et al.* ^16^, and chdlodds and chdloddsse are genetic associations with coronary heart disease risk for the same variants. These data variables are provided with the package. n is the sample size for the genetic associations. In practice n could be the sample size either for the exposure or the outcome, the smaller value is recommended to get appropriately-sized confidence intervals; see reference for detailed discussions ^14^.

The mr_mvcML function takes an *MRMVInput* object as input, created using the mr_mvinput command. The syntax is:


mr_mvcML(mr_mvinput(bx = cbind(ldlc, hdlc, trig),
   bxse = cbind(ldlcse, hdlcse, trigse),
   by = chdlodds, byse = chdloddsse), n = 17723)

where hdlc and hdlcse are genetic associations with HDL cholesterol and their standard errors, trig and trigse are genetic associations with triglycerides and their standard errors for the same 28 variants, and n is the sample size for the genetic associations with the exposures (or outcome if smaller). Again, these data variables are provided with the package.

The main options for these methods are DP = TRUE, which performs data perturbation and generally results in wider confidence intervals, but is recommended to provide appropriately-sized confidence intervals; MA = TRUE (for mr_cML), which performs model averaging, which again is recommended to provide appropriately-sized confidence intervals; and rho_mat (for mr_mvcML), which specifies the exposure correlation matrix between the summarized data for the exposures and outcome. If this is unspecified, then it is taken as the identity matrix, implying that the summarized data for the exposures and outcome are independent (typically because they were estimated in non-overlapping samples). Other options are provided to change the settings of the optimization functions used in the methods.

***Debiased inverse-variance weighted method.*** The mr_divw function performs the debiased inverse-variance weighted method ^17^. This method is an extension of the inverse-variance weighted (IVW) method that is more robust to weak instruments, with better bias and coverage properties.

The mr_divw function takes an *MRInput* object as input, created using the mr_input command. The syntax is:


mr_divw(mr_input(ldlc, ldlcse, chdlodds, chdloddsse))

The main options for this method are over.dispersion = TRUE, which allows for overdispersion in the variant-specific estimates (similar to a random-effects model for the IVW method), and diagnostics = FALSE, which provides a quantile—quantile plot of the variant-specific estimates, as a visual inspection for overdispersion and outliers.

***Penalized inverse-variance weighted method.*** The mr_pivw function performs the penalized inverse-variance weighted method ^18^. This method is an extension of the IVW method and the debiased inverse-variance weighted method, which handles weak instrument bias by a penalized log-likelihood function, and handles balanced horizontal pleiotropy by accounting for overdispersion in the variant-specific estimates.

The mr_pivw function takes an *MRInput* object as input, created using the mr_input command. The syntax is:


mr_pivw(mr_input(ldlc, ldlcse, chdlodds, chdloddsse))

The main options for this method are: lambda = 1, which is the penalty parameter. It plays a role in the bias-variance trade-off of the estimator. It is recommended to choose lambda = 1 to achieve the smallest bias and valid statistical inference; over.dispersion = TRUE, which allows for overdispersion in the variant-specific estimates; and delta = 0, which is a z-score threshold used for screening out weak instruments. delta should be greater than or equal to zero. When delta = 0, all variants provided will be used in the analysis. When delta > 0, the option sel.pval should be specified, which is the p-values of the genetic associations on the exposure. Then, the variants with sel.pval > 2*pnorm(delta,lower.tail = FALSE) will be removed from the analysis. The final option is Boot.Fieller = TRUE, which provides the p-value and the confidence interval of the causal effect calculated by the bootstrapping Fieller method.

***Generalized method of moments method.*** The mr_mvgmm function performs the generalized method of moments (GMM) method ^19^ for the multivariable Mendelian randomization setting. The key advantage of the GMM method is that estimates are more robust to weak instrument bias and/or measurement error in the exposures ^20^ compared with the standard IVW method, which is equivalent to a two-stage least squares approach with individual-level data ^7^. Weak instrument bias is particularly important in the multivariable setting, as bias in the multivariable setting can be in any direction, particularly if instrument strength varies between the exposures.

Unlike the multivariable IVW method ( mr_mvivw), the multivariable GMM method requires the sample sizes for genetic associations with the exposure, and the sample size for the genetic associations with the outcome. The method offers inferences that are robust to overdispersion in the variant-specific estimates (using the default option robust = TRUE).

If a genetic correlation matrix is not supplied in the mr_mvinput function, then the genetic variants will be assumed to be uncorrelated. There is also the option to use correlated variants if a genetic correlation matrix is supplied. If a genetic correlation matrix is supplied, the orientation of variants in the genetic correlation matrix ( *i.e.* the assumed effect alleles) should be harmonized with the summary statistics used in the analysis as described in the mr_pcgmm section below.

The syntax for the mr_mvgmm function is:


mr_mvgmm(mr_mvinput(bx = cbind(ldlc, hdlc, trig), bxse = cbind(ldlcse, hdlcse, trigse), by = chdlodds, byse = chdloddsse), nx=rep(17723,3), ny=17723)

***Principal component generalized method of moments methods.*** The mr_pcgmm and mr_mvpcgmm functions perform the principal component generalized method of moments (PC-GMM) method, for the univariable Mendelian randomization ( mr_pcgmm) and multivariable Mendelian randomization ( mr_mvpcgmm) settings ^21^. This method is very similar to the GMM method, the key difference being that the GMM method uses individual variants as instruments rather than principal components.

These methods are designed for use when performing Mendelian randomization using genetic variants from a single gene region ^22^. As an alternative to pruning and clumping approaches, which take a large number of variants from a gene region (potentially hundreds or thousands) and select a small number of uncorrelated (or weakly correlated) variants, the principal components approach performs dimension reduction on the full set of variant associations. The aim is to construct a small number of principal components which capture the information in the data, allowing the analysis to be performed using all available data, but avoiding numerical issues that would occur if highly-correlated genetic variants were included in the analysis. Previous investigations have shown that dimension reduction approaches can give additional precision compared with approaches using a small number of selected variants, and are less sensitive to variability arising from the variant selection process ^23^. Results from dimension reduction approaches are also less sensitive to misspecification of the variant correlation matrix, compared with other approaches using highly-correlated variants ^23, 24^.

Compared with other dimension reduction approaches that have been proposed ^25^, the PC-GMM method has some important advantages: it uses the Continuously Updating Generalized Method of Moments method ^26^, which provides estimates that are more robust to weak instruments than some other instrumental variable methods ^20^; and it can allow for overdispersion in the variant-specific estimates (using the default option robust = TRUE), which is recommended to provide appropriately-sized confidence intervals and valid inferences.

The syntax for the univariable mr_pcgmm function is:


mr_pcgmm(mr_input(bx = calcium, bxse = calciumse,
   by = fastgluc, byse = fastglucse, correlation = calc.rho),
   nx=6351, ny=133010)


where calcium, calciumse, fastgluc, fastglucse, and calc.rho are genetic association data on six correlated variants from the *CASR* gene region provided with the package and their associations with serum calcium levels ( calcium and associated standard errors calciumse) and fasting glucose levels ( fastgluc and associated standard errors fastglucse), nx is the sample size for genetic associations with the exposure, and ny is the sample size for genetic associations with the outcome. These associations are:

> mr_input(bx = calcium, bxse = calciumse,
>    by = fastgluc, byse = fastglucse, correlation = calc.rho)

    SNP exposure.beta exposure.se outcome.beta outcome.se
1 snp_1       0.00625     0.00233      0.02805     0.0122
2 snp_2       0.00590     0.00338      0.00953     0.0198
3 snp_3       0.01822     0.00318      0.03646     0.0173
4 snp_4       0.00598     0.00233      0.01049     0.0119
5 snp_5       0.00825     0.00229      0.02357     0.0122
6 snp_6       0.00651     0.00352      0.00204     0.0179

The syntax for the multivariable mr_mvpcgmm function is:


mr_mvpcgmm(mr_mvinput(bx = cbind(ldlc, hdlc, trig),
   bxse = cbind(ldlcse, hdlcse, trigse),
   by = chdlodds, byse = chdloddsse,
   correlation = diag(length(ldlc))),
   nx=rep(17723,3), ny=17723)


We note that this example does not use genetic variants from a single gene region, and so does not represent a recommended use case for the method. It is provided to demonstrate the code syntax.

The default operation of the mr_pcgmm and mr_mvpcgmm functions chooses the number of principal components to explain 99.9% of the variability in a weighted version of the variant correlation matrix. This can be varied, either by setting a different threshold (default thres = 0.999 corresponds to 99.9%) or by fixing the number of principal components using the r option (for example, r = 10 would select 10 principal components). As for the mr_mvcML function, the mr_mvpcgmm function requires the exposure correlation matrix, set using the cor.x option (although for mr_mvpcgmm, correlations with the outcome are not needed). If not specified, this is set to the identity matrix, implying that the summarized data for the exposures are estimated in non-overlapping samples. If these associations were estimated in the same dataset and these correlations are not known, a sensitivity analysis may be worthwhile.

***Correlated variants.*** Most methods in the MendelianRandomization package assume that all genetic variants are uncorrelated (see Table 1). The mr_ivw and mr_egger functions (and their multivariable counterparts) can account for correlations between variants, as can the mr_mvgmm and related methods ( mr_pcgmm and mr_mvpcgmm) introduced above. While using partly correlated variants can provide additional precision compared with using uncorrelated (or minimally-correlated) variants, there are several cautions to the use of correlated variants in Mendelian randomization.

First, the estimate from the analysis with correlated variants should not be strikingly more precise than the result with the lead variant only ( *i.e.* the variant having the lowest p-value in its association with the exposure) or with minimally-correlated variants. We would expect the correlated variants to explain slightly more variance in the exposure, and so we would expect the standard error to reduce and confidence intervals to narrow slightly when using correlated variants. However, if the standard error when using correlated variants is much smaller than when using the lead variant only (say, it is two or three times smaller), then inputs should be checked carefully, as the increase in precision may represent a convergence issue. Reporting a sensitivity analysis using fewer variants or principal components is recommended.

Second, many software tools report the squared-correlation matrix (the r ^2^ matrix) between variants, not the (signed) correlation matrix. Mendelian randomization requires the correlation matrix, which provides correlations between variants.

Third, the signs of the correlations (positive or negative) must be correctly specified. If the correlation matrix is calculated using the same effect and non-effect alleles as the summarized data, then the correlations should have the correct signs ^27^. If not, then the correlation matrix should be harmonized to the same effect alleles by flipping signs in the relevant rows and columns. Failure to harmonize the genetic correlation matrix can result in erroneous estimates. This can be implemented in R by adapting the following example code:


flip = c(+1, +1, -1, +1, -1, +1)
calc.rho.signed = calc.rho*flip%o%flip


where flip is +1 for variants for which the alleles are correctly aligned, and -1 for variants for which the alleles are incorrectly aligned, and the elements of flip correspond to the genetic variants in order.

In this example, before orientation, the variant correlation matrix is:

> calc.rho

       [,1]   [,2]   [,3]   [,4]   [,5]   [,6]
[1,]  1.000  0.070  0.094 -0.172  0.079 -0.104
[2,]  0.070  1.000 -0.050  0.081 -0.080 -0.014
[3,]  0.094 -0.050  1.000 -0.306  0.349 -0.134
[4,] -0.172  0.081 -0.306  1.000 -0.129  0.446
[5,]  0.079 -0.080  0.349 -0.129  1.000 -0.289
[6,] -0.104 -0.014 -0.134  0.446 -0.289  1.000


After orientation, the variant correlation matrix is:

> calc.rho.signed

       [,1]   [,2]   [,3]   [,4]   [,5]   [,6]
[1,]  1.000  0.070 -0.094 -0.172 -0.079 -0.104
[2,]  0.070  1.000  0.050  0.081  0.080 -0.014
[3,] -0.094  0.050  1.000  0.306  0.349  0.134
[4,] -0.172  0.081  0.306  1.000  0.129  0.446
[5,] -0.079  0.080  0.349  0.129  1.000  0.289
[6,] -0.104 -0.014  0.134  0.446  0.289  1.000


As the third and fifth elements of flip are -1 (and all others are +1), all entries in the third row or column, and the fifth row or column, have flipped in sign (from positive to negative, or from negative to positive). For example, the third entry in the first row was +0.094, but is now -0.094. The element in the third row and fifth column (+0.349) is flipped in sign twice, and so its sign ends up unchanged; similarly for the element in the third row and third column, the fifth row and third column, and so on.

Fourth, the correlation matrix should be obtained from the same population (or same ancestry group) as the original data, to avoid mismatches between the correlation matrix and the summarized data. If the exposure and outcome data are from different ancestry groups, then correlated variant analyses should not be attempted. If the exposure and outcome data are from the same ancestry group, then correlations should be taken from the outcome data by preference, but either (or use of a suitable reference population) is acceptable.

Fifth, analysts should consider pruning out extremely highly correlated variants, as these are likely to contribute little to the analysis, even when using the mr_pcgmm and mr_mvpcgmm methods. A suggestion when using these methods is to take pairs of variants that are correlated at r ^2^ > 0.95, and remove one of the pair at random until no such pairs remain. This can be implemented using the code below for a correlation matrix rho:

set.seed(496)         # for reproducibility
thres = sqrt(0.95)    # threshold set to r2>0.95
omit = NULL           # set up list of variants to be omitted
rho.upper = rho       # correlation matrix
rho.upper[lower.tri(rho, diag=TRUE)] <- 0
                      # only consider upper triangle of correlations
j=1                   # set counter to 1

while (max(abs(rho.upper), na.rm=TRUE)> thres) {
 omit[j] = ifelse(rbinom(1, 1, 0.5)==1, 
   which.max(apply(abs(rho.upper), 1, max, na.rm=TRUE)), 
   which.max(apply(abs(rho.upper), 2, max, na.rm=TRUE)))
                      # find the highest correlation value
                      # select either the row or column at random
                      # add this to the list of omitted variants
 rho.upper[omit[j],] <- 0 # set the correlations in this row to zero
 rho.upper[,omit[j]] <- 0 # set the correlations in this column to zero
                          # (to avoid selecting the same variant again)
 j=j+1                # increment the counter 
 }  # stop when no more pairwise correlations exceed threshold

For other methods that allow for correlated variants, either pruning at a stricter threshold (say, r ^2^ < 0.3) or applying another approach for variant selection (such as fine-mapping or conditional modelling) is recommended to avoid high levels of variant multicollinearity.

Following these steps should ensure that the correlation matrix is relevant to the data under analysis, is correctly harmonized, and does not include pairs of variants with very highly correlations.

***Variant correlation matrix and exposure correlation matrix.*** We note the distinction between the variant correlation matrix which represents correlations between variant association estimates occurring due to linkage disequilibrium, and the exposure correlation matrix which represents correlations between variant association estimates occurring due to correlations in exposure measurements, which arise if these estimates are obtained in the same individuals. If the analyst has access to individual-level data on allele counts, the variant correlation matrix can be calculated directly using the correlation ( cor) function in R applied to the matrix of allele counts. The variant correlation matrix can be obtained from a reference population; for example, correlations for European ancestry individuals from UK Biobank are available here, and correlations for other ancestry groups (although for much smaller sample sizes) are available at https://ldlink.nci.nih.gov/?tab=ldmatrix (although note these are squared correlations, so not suitable for direct use in the MendelianRandomization package functions). 

The exposure correlation matrix can only be estimated from individual-level data, and hence a sensitivity analysis for its value is suggested if it is unknown. However, investigations have indicated that estimates are generally insensitive to the correct specification of the exposure correlation matrix, and so the default value of the identity matrix is likely to be a reasonable choice. A sensitivity analysis varying this matrix is recommended, to assess whether findings change for different values of this matrix.

***Operation.*** The R software environment (RRID:SCR_001905) runs on a wide variety of UNIX platforms, Windows, and MacOS, and requires minimal computer resources (256 kilobytes of RAM is recommended). The package requires R version 3.3.0 or higher. As the package now uses C++ code, an up-to-date version of Rtools should be installed to successfully install the package. This current work used R version 4.3.1.

## Use case

### Instrument strength

A further update to the MendelianRandomization package is the functionality to report F statistics in the mr_ivw and mr_mvivw commands. The first-stage F statistic is a measure of the variance in the exposure explained by the genetic variants in the exposure dataset ^28^. It is roughly equal to the R ^2^ statistic (the proportion of variance explained) multiplied by the sample size and divided by the number of instruments. Larger F statistics correspond to stronger instruments. The F statistic cannot be determined exactly based on summary statistics, but the simple formula implemented in the package provides a good approximation. While the mythical threshold of 10 for F statistics ^29^ is an oversimplification, this value does provide a reasonable yardstick for judging the degree of potential bias due to weak instruments (although we would strongly caution against basing an analysis plan on measured values of the F statistic in the dataset under analysis ^28^). We note that although weak instrument bias is typically in the direction of the null for two-sample univariable Mendelian randomization ^30^, this is not necessarily the case for two-sample multivariable Mendelian randomization ^31^.

The default implementation of the mr_ivw and mr_mvivw functions assumes that genetic variants are uncorrelated. Correlations can be specified in the mr_input or mr_mvinput command:


mr_ivw(mr_input(calcium, calciumse,
   fastgluc, fastglucse, corr=calc.rho))


If two uncorrelated variants both explain 3% of the variance in the exposure, then together they explain 6% of the variance in the exposure. If two correlated variants both explain 3% of the variance in the exposure, then the variance explained by both variants could be as low as 3% (if the variants are perfectly correlated) or as high as 6% (if the correlation is negligible), or a value between these two, depending on the magnitude of correlation. This correlation similarly affects estimates of the F statistic. When the variant correlation matrix is specified, the mr_ivw function accounts for these correlations in the calculation of the F statistic using a formula that involves the inverse of the Cholesky transform of the correlation matrix ^32^.

For multivariable Mendelian randomization, the key measure of instrument strength is not the univariable F statistic for each exposure separately, but the conditional F statistic, representing the independent proportion of variance explained in each exposure, after accounting for associations with the other exposures ^33, 34^. This is because if the genetic associations with one exposure were strong, but were near-perfectly correlated with the genetic associations with another exposure, then the multivariable model would not be able to differentiate between the effects of the two exposures.

The mr_mvivw function calculates estimates of the conditional F statistics based on summarized data. In order to identify and reliably estimate multivariable exposure effects through Mendelian randomization, we require that the genetic predictors of the exposures are not collinear. Hence, conditional F-tests assess whether the genetic predictors of any one exposure can be expressed as a linear combination of genetic predictors of other exposures in the model. Low values of conditional F-test statistics for a given exposure suggest that the exposure may not be identified, and hence estimates may suffer from weak instrument bias. This is generally a bigger concern for two-sample multivariable, rather than two-sample univariable, Mendelian randomization analyses since weak instrument biases in estimation are not necessarily toward the null of no causal effect, and hence inferences can suffer from inflated type I error rates (false positive findings). This calculation of conditional F-statistics involves the sample size for the genetic associations with the exposures, and conditional F statistics are only calculated if these sample sizes are provided. If a single value is provided for the sample size, then it is assumed that this sample size holds for all exposures. For example, the code:


mr_mvivw(mr_mvinput(bx = cbind(ldlc, hdlc, trig),
   bxse = cbind(ldlcse, hdlcse, trigse),
   by = chdlodds, byse = chdloddsse), nx = 17723)


gives output:


Number of Variants : 28 
------------------------------------------------------------------
   Exposure Estimate Std Error  95% CI       p-value Cond F-stat
 exposure_1    1.925     0.439  1.064, 2.786   0.000        20.3
 exposure_2   -0.590     0.555 -1.677, 0.498   0.288        12.9
 exposure_3    0.723     0.230  0.272, 1.174   0.002        13.3
------------------------------------------------------------------

By comparison, the code:


mr_ivw(mr_input(ldlc, ldlcse, chdlodds, chdloddsse))


gives output including:


F statistic = 28.0.


We see that the univariable F statistic for LDL-cholesterol is 28.0, but the conditional F statistic is 20.3. Similarly, the univariable F statistic for HDL-cholesterol is 27.6, but the conditional F statistic is 12.9, and for triglycerides, the univariable F statistic is 41.6, but the conditional F statistic is 13.3. The instrument strength for LDL-cholesterol is similar in the multivariable analysis compared to the univariable analyses, but for HDL-cholesterol and triglycerides the instrument strength is weakened considerably.

F statistics and conditional F statistics are also provided by the mr_pcgmm and mr_mvpcgmm functions, as these may help the user to judge how varying the number of principal components selected affects instrument strength.

## Conclusions

In summary, the MendelianRandomization package has added a number of features since the 0.5.0 version: to implement additional estimation methods, to implement methods for highly correlated variants, and to report F statistics.

We conclude by again repeating the warning that we stated at the end of the manuscript accompanying the initial package release ^11^: while this software simplifies the operational aspects of a Mendelian randomization, the truly difficult parts of an analysis are choosing sensible risk factors and outcomes, selecting genetic variants that are plausible instrumental variables, performing a reasonable range of analyses, and interpreting the results with care and caution ^13^. These aspects of an analysis cannot be automated ^35^.

## Data Availability

Zenodo. MendelianRandomization package version 0.9.0. https://doi.org/10.5281/zenodo.8305056 ^36^. This project contains the following underlying data: Folder/Files containing input data used in the “use case section”.

## Software availability

**Software available from:** https://cran.r-project.org/web/packages/MendelianRandomization/index.html.

**Archived source code at time of publication:** http://doi.org/10.5281/zenodo.8305056 ^36^

License: AGPL-3.0-only
