# Supplementary material for: MendelianRandomization v0.9.0: updates to an R package for performing Mendelian randomization analyses using summarized data
Source: Wellcome Open Res. 2023 Nov 21;8:449. Originally published 2023 Oct 12. [Version 2] doi: 10.12688/wellcomeopenres.19995.2 (PMC10616660; doi:10.12688/wellcomeopenres.19995.2)
Supplement: Peer review history

# Reviewer report: Reviewer response for version 2

**DOI:** 10.21956/wellcomeopenres.22678.r70304

**Published:** 2023-11-30

**Authors:** Hajime Yamazaki

**Affiliations:** 1 Section of Clinical Epidemiology, Department of Community Medicine, Graduate School of Medicine, Kyoto University, Kyoto, Japan

No further comments.

Are the conclusions about the tool and its performance adequately supported by the findings presented in the article?

Yes

Is the rationale for developing the new software tool clearly explained?

Yes

Is the description of the software tool technically sound?

Yes

Are sufficient details of the code, methods and analysis (if applicable) provided to allow replication of the software development and its use by others?

Yes

Is sufficient information provided to allow interpretation of the expected output datasets and any results generated using the tool?

Yes

Reviewer Expertise:

Clinical epidemiology, Gastroenterology

I confirm that I have read this submission and believe that I have an appropriate level of expertise to confirm that it is of an acceptable scientific standard.

# Reviewer report: Reviewer response for version 2

**DOI:** 10.21956/wellcomeopenres.22678.r70305

**Published:** 2023-11-23

**Authors:** Ricardo Costeira

**Affiliations:** 1 Department of Twin Research and Genetic Epidemiology, King's College London, London, England, UK

No further comments.

Are the conclusions about the tool and its performance adequately supported by the findings presented in the article?

Yes

Is the rationale for developing the new software tool clearly explained?

Yes

Is the description of the software tool technically sound?

Yes

Are sufficient details of the code, methods and analysis (if applicable) provided to allow replication of the software development and its use by others?

Yes

Is sufficient information provided to allow interpretation of the expected output datasets and any results generated using the tool?

Yes

Reviewer Expertise:

Genomics

I confirm that I have read this submission and believe that I have an appropriate level of expertise to confirm that it is of an acceptable scientific standard.

# Response

**Published:** 2023-11-23

**Authors:** Stephen Burgess

**Affiliations:** University of Cambridge Addenbrooke's Hospital Cambridge, UK

Thank you Ricardo for the fast and positive response, and for your previous comments on this article, which have helped improve the work.

# Reviewer report: Reviewer response for version 1

**DOI:** 10.21956/wellcomeopenres.22141.r68639

**Published:** 2023-10-30

**Authors:** Hajime Yamazaki

**Affiliations:** 1 Section of Clinical Epidemiology, Department of Community Medicine, Graduate School of Medicine, Kyoto University, Kyoto, Japan

This article describes the updated functions of the MendelianRandomization package, which is widely used to conduct Mendelian randomization analyses. I believe that the updated functions and their detailed descriptions in this article will be immensely beneficial for researchers.

 To enhance the readability and comprehensibility of the article, I propose the following suggestions:

1. At the beginning of the Methods section, the authors introduce ‘three instrumental variable assumptions.’ It would be prudent to both explicate these assumptions in the Introduction section and explain why genetic variants can serve as a proxy for random assignment.
2. This article utilizes data pertaining to LDL cholesterol, HDL cholesterol, triglycerides, and coronary heart disease risk as an illustrative example. It would be beneficial to clearly articulate the research question associated with this example, incorporate a graphical representation to delineate the relationship between genetic variants and these variables, and provide a brief overview of the selection process of these genetic variants within a multivariable Mendelian randomization context.
3. It would be beneficial to include a small portion of the example dataset, comprising the correlation matrix, within the article along with a reference to its availability in the MendelianRandomization package.
4. The authors draw a distinction between the variant correlation matrix and the exposure correlation matrix. A more detailed explanation on how to estimate each of these, as well as guidance on which correlation matrix is pertinent for use with the MendelianRandomization package, would be beneficial.
5. The “Principal Component Generalized Method of Moments” section is extensive and could be made more readable by dividing it into two distinct subsections, each with its own subheading.

Are the conclusions about the tool and its performance adequately supported by the findings presented in the article?

Yes

Is the rationale for developing the new software tool clearly explained?

Yes

Is the description of the software tool technically sound?

Yes

Are sufficient details of the code, methods and analysis (if applicable) provided to allow replication of the software development and its use by others?

Yes

Is sufficient information provided to allow interpretation of the expected output datasets and any results generated using the tool?

Yes

Reviewer Expertise:

Clinical epidemiology, Gastroenterology

I confirm that I have read this submission and believe that I have an appropriate level of expertise to confirm that it is of an acceptable scientific standard.

# Response

**Published:** 2023-11-12

**Authors:** Stephen Burgess

**Affiliations:** University of Cambridge Addenbrooke's Hospital Cambridge, UK

**Response to reviewers for “MendelianRandomization v0.9.0: updates to an R package for performing Mendelian randomization analyses using summarized data”**

 We would like to express thanks to the reviewers for their time and comments. Replies to points are indicated by angle brackets, and changes to the paper as a result of these comments are clearly indicated. We have numbered the comments for reference as A0, A1, A2, … for the first reviewer; and B0, B1, B2, … for the second reviewer.

*Reviewer 2:* Hajime Yamazaki, Section of Clinical Epidemiology, Department of Community Medicine, Graduate School of Medicine, Kyoto University, Kyoto, Japan

 B0. This article describes the updated functions of the MendelianRandomization package, which is widely used to conduct Mendelian randomization analyses. I believe that the updated functions and their detailed descriptions in this article will be immensely beneficial for researchers.

 > We thank the reviewer for their kind comments and positive view of their manuscript. <

 To enhance the readability and comprehensibility of the article, I propose the following suggestions:

 B1. At the beginning of the Methods section, the authors introduce ‘three instrumental variable assumptions.’ It would be prudent to both explicate these assumptions in the Introduction section and explain why genetic variants can serve as a proxy for random assignment. > We have added a brief description of the three classical instrumental variable assumptions required to identify a causal effect, as well as a brief motivation as to why genetic variants are often suitable candidate instrumental variables. <

 B2. This article utilizes data pertaining to LDL cholesterol, HDL cholesterol, triglycerides, and coronary heart disease risk as an illustrative example. It would be beneficial to clearly articulate the research question associated with this example, incorporate a graphical representation to delineate the relationship between genetic variants and these variables, and provide a brief overview of the selection process of these genetic variants within a multivariable Mendelian randomization context.

 > We have added a brief description of how these genetic variants were chosen. While we appreciate the reviewer’s point, we would not want to give the impression that the data included in the Mendelian randomization package are anything other than illustrative. They are not the ideal data to optimally address any research question. <

 B3. It would be beneficial to include a small portion of the example dataset, comprising the correlation matrix, within the article along with a reference to its availability in the MendelianRandomization package.

 > We have added the calcium and fasting glucose data to the manuscript, as well as their correlation matrix (see also point A2). <

 B4. The authors draw a distinction between the variant correlation matrix and the exposure correlation matrix. A more detailed explanation on how to estimate each of these, as well as guidance on which correlation matrix is pertinent for use with the MendelianRandomization package, would be beneficial. > There are various tools to estimate the variant correlation matrix. If you have access to individual-level genetic data (that is, allele counts for each SNP), you can calculate the variant correlation matrix using the correlation ( *cor*) function in R. Alternatively, some pre-computed correlation matrices are available for download, for example using the LDlink webtool ( https://ldlink.nci.nih.gov/?tab=ldmatrix) or the ld_extract function in the TwoSampleMR package. Variant correlation matrices for UK Biobank participants of European ancestries are available at https://aws.amazon.com/marketplace/pp/prodview-4bhcvjnh4b4cs#resources. > We have listed some of these sources in the manuscript. > Exposure correlation estimates are required by some methods when genetic associations with any two exposures are measured from the same sample. Exposure correlation estimates may be more difficult to obtain than variant correlation estimates, but they could be estimated using individual-level data on the exposures from the same sample or an external sample used to measure genetic variant–exposure associations. In general, we have found estimation and inferences to be quite insensitive to mis-specified exposure correlations, and in practice we would recommend performing a sensitivity analysis that varies the inputted exposure correlations to assess sensitivity of findings to the specification of this matrix.

 > We have expanded the discussion about exposure correlation matrices in the manuscript. <

 B5. The “Principal Component Generalized Method of Moments” section is extensive and could be made more readable by dividing it into two distinct subsections, each with its own subheading.

 > We have added separate sections marked “Correlated variants” and “Variant correlation matrix and exposure correlation matrix” that includes much of the text previously included in the “Principal Component Generalized Method of Moments” section, and hence breaking up this previously over-long section. <

# Reviewer report: Reviewer response for version 1

**DOI:** 10.21956/wellcomeopenres.22141.r68641

**Published:** 2023-10-30

**Authors:** Ricardo Costeira

**Affiliations:** 1 Department of Twin Research and Genetic Epidemiology, King's College London, London, England, UK

In this manuscript, the authors describe updates to the MendelianRandomization package implemented in R. In the package’s latest version (v0.9.0), users can find functions to perform newer robust methods of MR (such as debiased and penalised IVW MR), consider variant correlation, and obtain F-statistics to estimate the strength of instrument variables.

 The authors provide good rationale for when and how to implement the newer MR methods. Examples of code are abundant throughout the manuscript, and the package’s functions are explained in detail. The authors set guidelines for implementing genetic correlation in MR and highlight potential caveats of the analysis. Altogether, the manuscript is useful to understand the newer functions available in MendelianRandomization v0.9.0 and has valuable insights into the practical considerations of applying more complex MR methods. Interpretation of newer output (the F-statistic) is included.

**Minor comments:**

 Under the section of “Principal component generalized method of moments methods”:  

- “where calcium, calciumse, fastgluc, fastglucse, and calc.rho are data on six correlated variants from the CASR gene region provided with the package” – please reword to clarify the meaning of the calcium, calciumse, fastgluc and fastglucse variables.
- Is “flip = c(+1, +1, -1, +1, -1, +1)” length = 6 because “calc.rho” is a matrix of 6 variants? Please show “calc.rho” before and after applying the function “calc.rho*flip%o%flip”. This can help understand the structure and signs of “calc.rho” going into the MR analysis.
- Regarding the correlation matrix coming from linkage disequilibrium data or exposure correlation: “However, our investigations have indicated that estimates are generally insensitive to the correct specification of this matrix.” – Please expand on this comment.

Under the section “Instrument Strength”:

- “We note that although weak instrument bias is typically in the direction of the null for two-sample univariable Mendelian randomization, this is not necessarily the case for two-sample multivariable Mendelian randomization.” and “This is generally a bigger concern for two-sample multivariable, rather than two-sample univariable, Mendelian randomization analyses since weak instrument biases in estimation are not necessarily toward the null of no causal effect, and hence inferences can suffer from inflated type I error rates.” Please explain why this is the case. Additionally, please comment on how weak instrument bias can affect other types of MR.

Are the conclusions about the tool and its performance adequately supported by the findings presented in the article?

Yes

Is the rationale for developing the new software tool clearly explained?

Yes

Is the description of the software tool technically sound?

Yes

Are sufficient details of the code, methods and analysis (if applicable) provided to allow replication of the software development and its use by others?

Yes

Is sufficient information provided to allow interpretation of the expected output datasets and any results generated using the tool?

Yes

Reviewer Expertise:

Genomics

I confirm that I have read this submission and believe that I have an appropriate level of expertise to confirm that it is of an acceptable scientific standard.

# Response

**Published:** 2023-11-12

**Authors:** Stephen Burgess

**Affiliations:** University of Cambridge Addenbrooke's Hospital Cambridge, UK

We would like to express thanks to the reviewers for their time and comments. Replies to points are indicated by angle brackets, and changes to the paper as a result of these comments are clearly indicated. We have numbered the comments for reference as A0, A1, A2, … for the first reviewer; and B0, B1, B2, … for the second reviewer.

*Reviewer 1*: Ricardo Costeira, Department of Twin Research and Genetic Epidemiology, King's College London, London, England, UK

 A0. In this manuscript, the authors describe updates to the MendelianRandomization package implemented in R. In the package’s latest version (v0.9.0), users can find functions to perform newer robust methods of MR (such as debiased and penalised IVW MR), consider variant correlation, and obtain F-statistics to estimate the strength of instrument variables. The authors provide good rationale for when and how to implement the newer MR methods. Examples of code are abundant throughout the manuscript, and the package’s functions are explained in detail. The authors set guidelines for implementing genetic correlation in MR and highlight potential caveats of the analysis. Altogether, the manuscript is useful to understand the newer functions available in MendelianRandomization v0.9.0 and has valuable insights into the practical considerations of applying more complex MR methods. Interpretation of newer output (the F-statistic) is included.

 > We thank the reviewer for their kind comments and positive view of this manuscript. <

 Minor comments: Under the section of “Principal component generalized method of moments methods”: 

 A1. “where calcium, calciumse, fastgluc, fastglucse, and calc.rho are data on six correlated variants from the CASR gene region provided with the package” – please reword to clarify the meaning of the calcium, calciumse, fastgluc and fastglucse variables.

 > We have clarified that *calcium* represents the genetic associations with serum calcium levels, and *fastgluc* the genetic associations with fasting glucose levels ( *calciumse* and *fastglucse* are the standard errors respectively). <

 A2. Is “flip = c(+1, +1, -1, +1, -1, +1)” length = 6 because “calc.rho” is a matrix of 6 variants? Please show “calc.rho” before and after applying the function “calc.rho*flip%o%flip”. This can help understand the structure and signs of “calc.rho” going into the MR analysis.

 > We have clarified that the entries in the *flip* vector correspond to the six genetic variants in turn. We have added to the manuscript a representation of the correlation matrix before and after orientation. <

 A3. Regarding the correlation matrix coming from linkage disequilibrium data or exposure correlation: “However, our investigations have indicated that estimates are generally insensitive to the correct specification of this matrix.” – Please expand on this comment.

 > We have clarified that this sentence refers to the exposure correlation matrix. <

 Under the section “Instrument Strength”:

 A4. “We note that although weak instrument bias is typically in the direction of the null for two-sample univariable Mendelian randomization, this is not necessarily the case for two-sample multivariable Mendelian randomization.” and “This is generally a bigger concern for two-sample multivariable, rather than two-sample univariable, Mendelian randomization analyses since weak instrument biases in estimation are not necessarily toward the null of no causal effect, and hence inferences can suffer from inflated type I error rates.” Please explain why this is the case. Additionally, please comment on how weak instrument bias can affect other types of MR.

 > Weak instrument bias is analogous to classical measurement error bias in a regression model. With a single regressor, error in the regressor leads to underestimation of the regression coefficient; this is known as regression dilution bias. With multiple regressors, bias due to measurement error can be in any direction. For example, suppose we have two regressors that are highly correlated, one of which is a causal risk factor for the outcome, and the other is not. Further, one is measured with substantial error, whereas the other is measured with no error. In a regression model, the coefficient for the regressor measured with error tends towards zero regardless of which is truly the causal risk factor. This could lead the coefficient for the precisely measured exposure being either over- or underestimated, depending on its true value and the true value for the coefficient of the imprecisely measured regressor. In a prediction model, this may be of little consequence, as the predicted values of the outcome may be insensitive to the exposure coefficients – which is large, which is close to zero. However, if the model coefficients have a causal interpretation, then the coefficients matter, as the regressor with the non-zero coefficient is identified as the causal factor.

 > Generally speaking, bias towards the null in Mendelian randomization is of lesser consequence, as the primary goal of Mendelian randomization is not estimation. Bias towards the null may reduce power to detect a true causal effect, but it will not incorrectly suggest a false causal effect (which is regarded in the Neyman—Pearson hypothesis framework as a more serious error).

 > As this explanation is fairly involved, we do not think it is appropriate to include in this manuscript. However, given that this journal performs open peer review, it can be found by the interested reader. <
